# Supplementary material for: Switching of Photocatalytic Tyrosine/Histidine Labeling and Application to Photocatalytic Proximity Labeling
Source: Int J Mol Sci. 2022 Oct 2;23(19):11622. doi: 10.3390/ijms231911622 (PMC9569449; doi:10.3390/ijms231911622)
Supplement: Supplementary file 1 [file ijms-23-11622-s001.zip › ijms-1921671-supplementary.pdf]

Supporting Information

Switching of Photocatalytic Tyrosine/Histidine Labeling and Application to Photocatalytic Proximity Labeling

Keita Nakane[1], Haruto Nagasawa[1], Chizu Fujimura[2], Eri Koyanagi[2], Shusuke Tomosige[1], Minoru Ishikawa[1], Shinichi Sato[1,2]*

1. Graduate School of Life Sciences, Tohoku University, 2-1-1 Katahira, Aoba-ku, Sendai 980-8577, Japan
2. Frontier Research Institute for Interdisciplinary Sciences, Tohoku University, 2-1-1 Katahira, Aoba-ku, Sendai 980-8577, Japan

*Correspondence: shinichi.sato.e3@tohoku.ac.jp; Tel.: +81-22-217-6373

List of contents

1. Supporting Data 2–15

1. Supporting Data

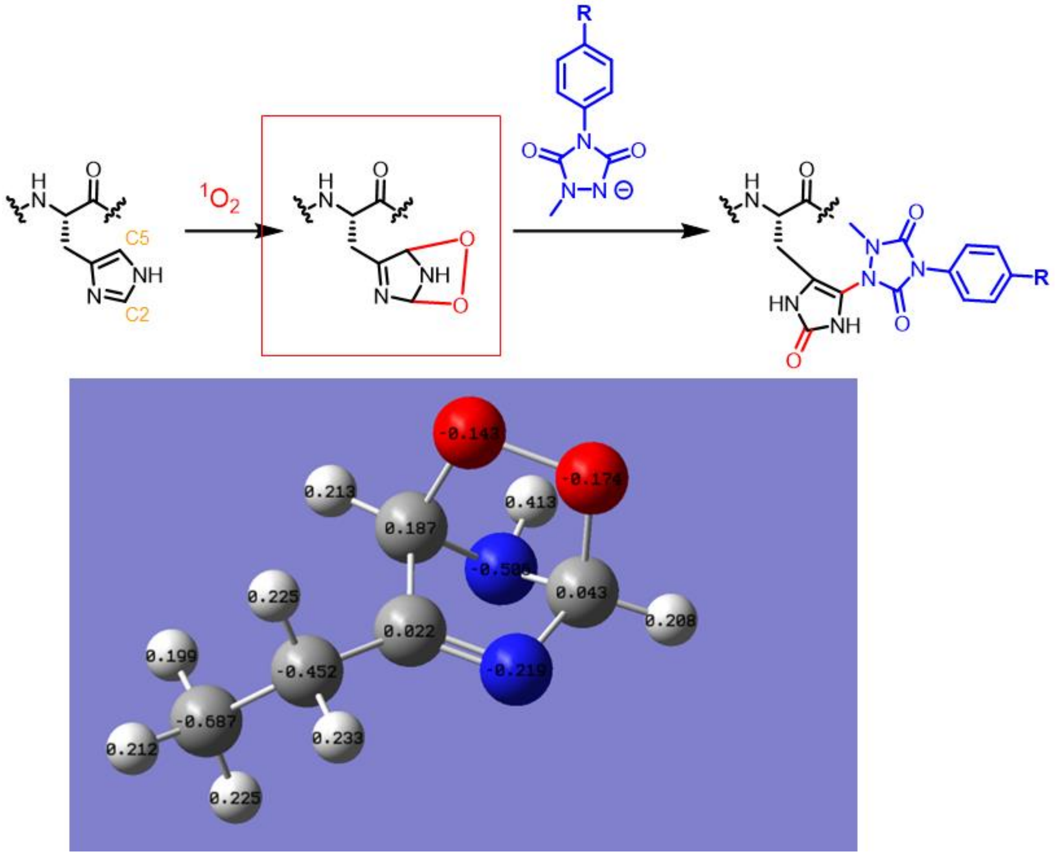

Figure S1. Mulliken charges of endoperoxide from histidine calculated by DFT method.

The electron-donating nature of two nitrogen atoms makes the C2 position less electrophilic than the C5 position. Gaussian calculations show that C5 has a positive charge at the Mulliken charge, suggesting that C5 is reactive.

An endoperoxide between the C4 and C2 positions is also possible. In that case, C4 of the endoperoxide is bulky and less reactive, and C2 is not reactive for the same reason as above.

In our previous report [26], we showed that the selective reaction occurs at the C5 position under the reaction conditions with $^1O_2$ by NMR analysis of a purified product.

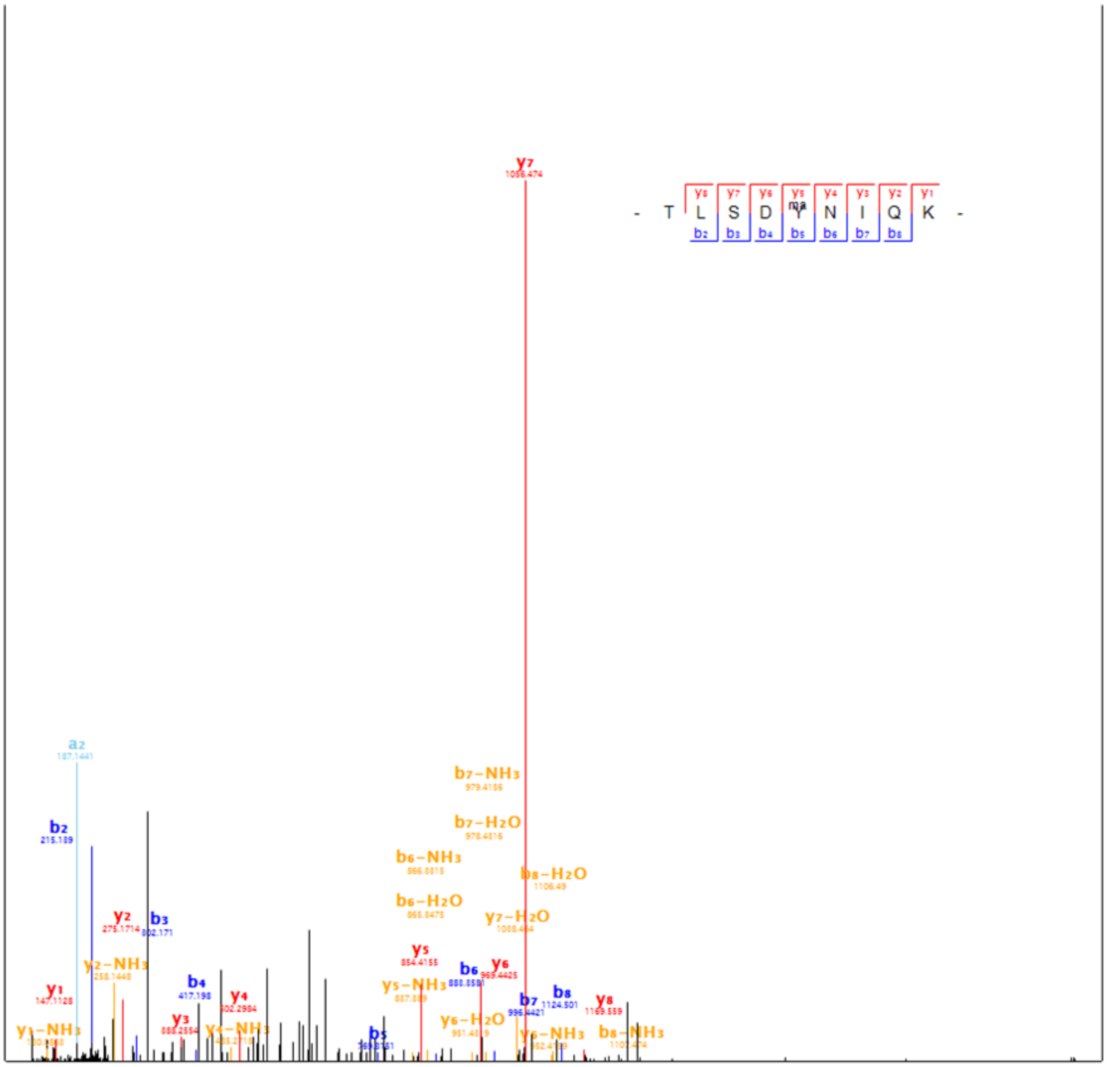

Figures S2. MS/MS analysis of the peak containing labeled Tyr (TLSDY*NIQK).

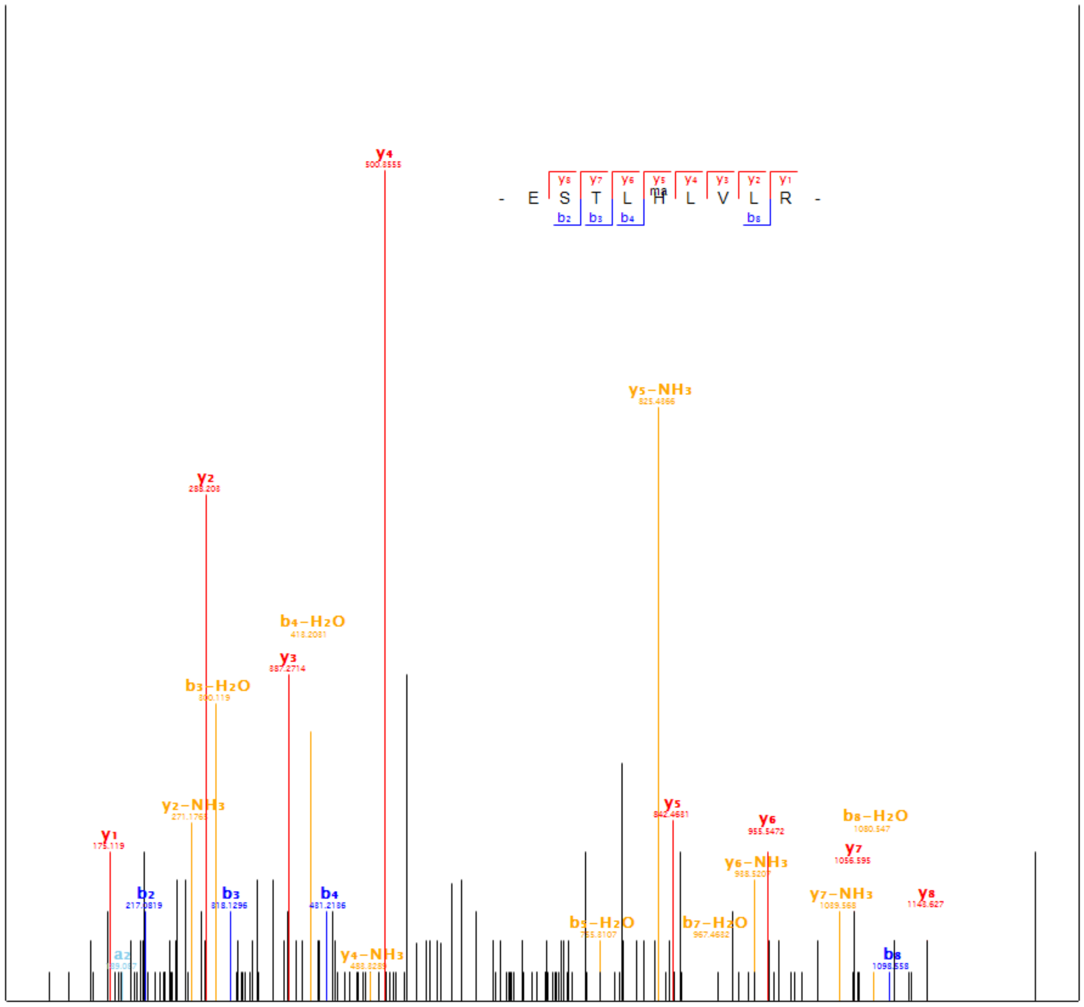

Figures S3. MS/MS analysis of the peak containing labeled His (ESTLH*LVLR).

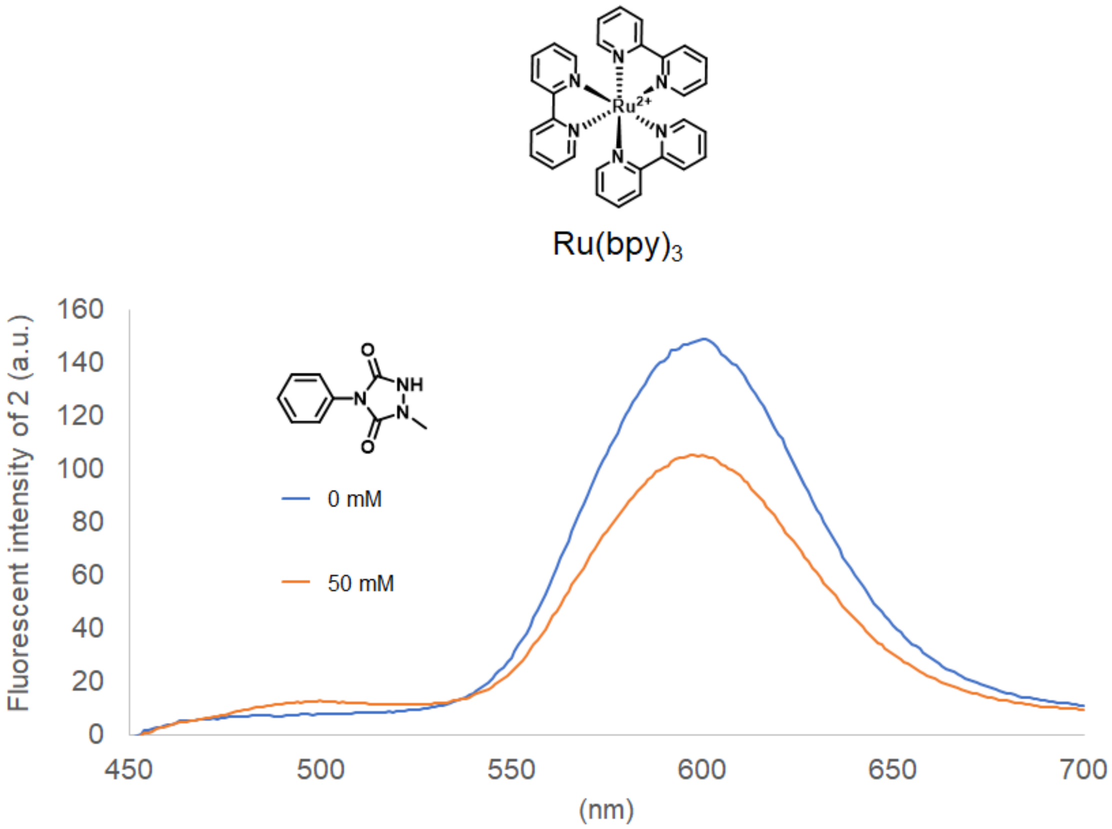

Figures S4. Stern-Volmer fluorescence quenching experiment. Compound **2** (50 nM, in 45% CH_3CN and 50% DMSO in 10 mM MES buffer (pH7.4)) with 0 or 50 mM **1**.

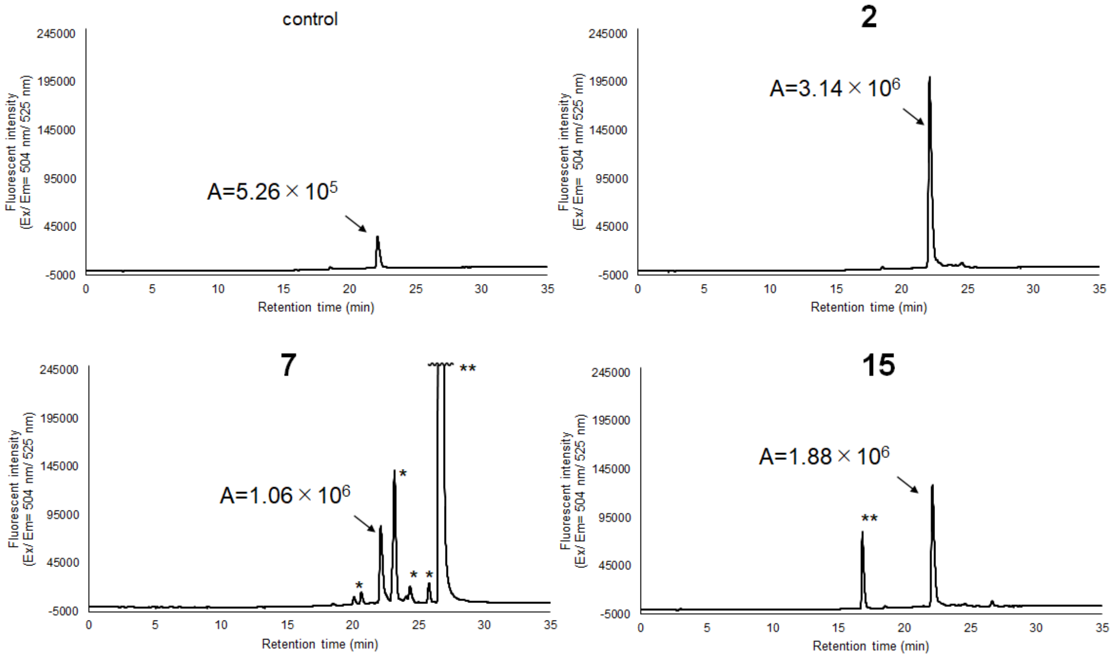

Figures S5. Detection of oxidized SOSG peak in each reaction condition by HPLC.

Arrows: oxidized SOSG by $^1O_2$.

*: oxidized/ decomposed dye. **: BODIOY (**7**) or ATTO465-CO_2H (**15**).

SOSG itself also produces singlet oxygen. Therefore, we evaluated the increase in fluorescence peak intensity of oxidized SOSG by each dye, considering the signal of SOSG alone as background.

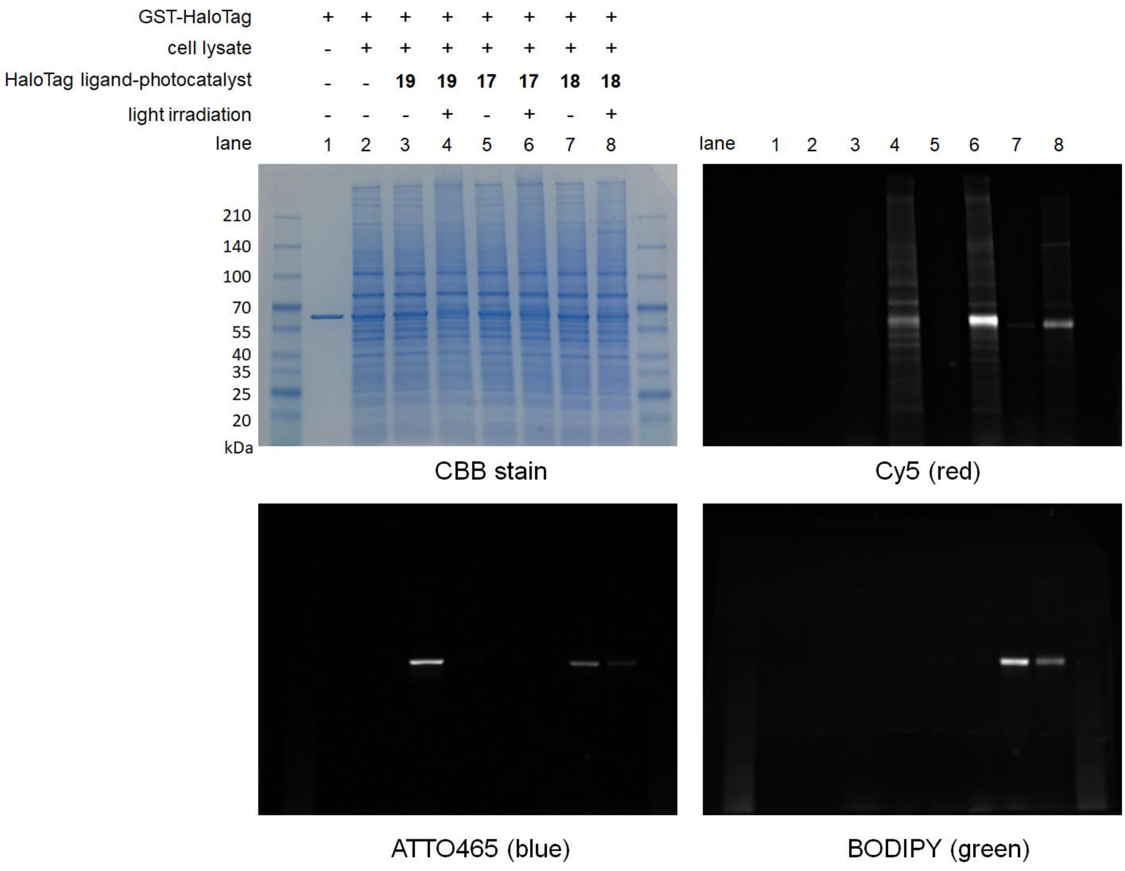

Figures S6. The gel image before processing of Figure 5.

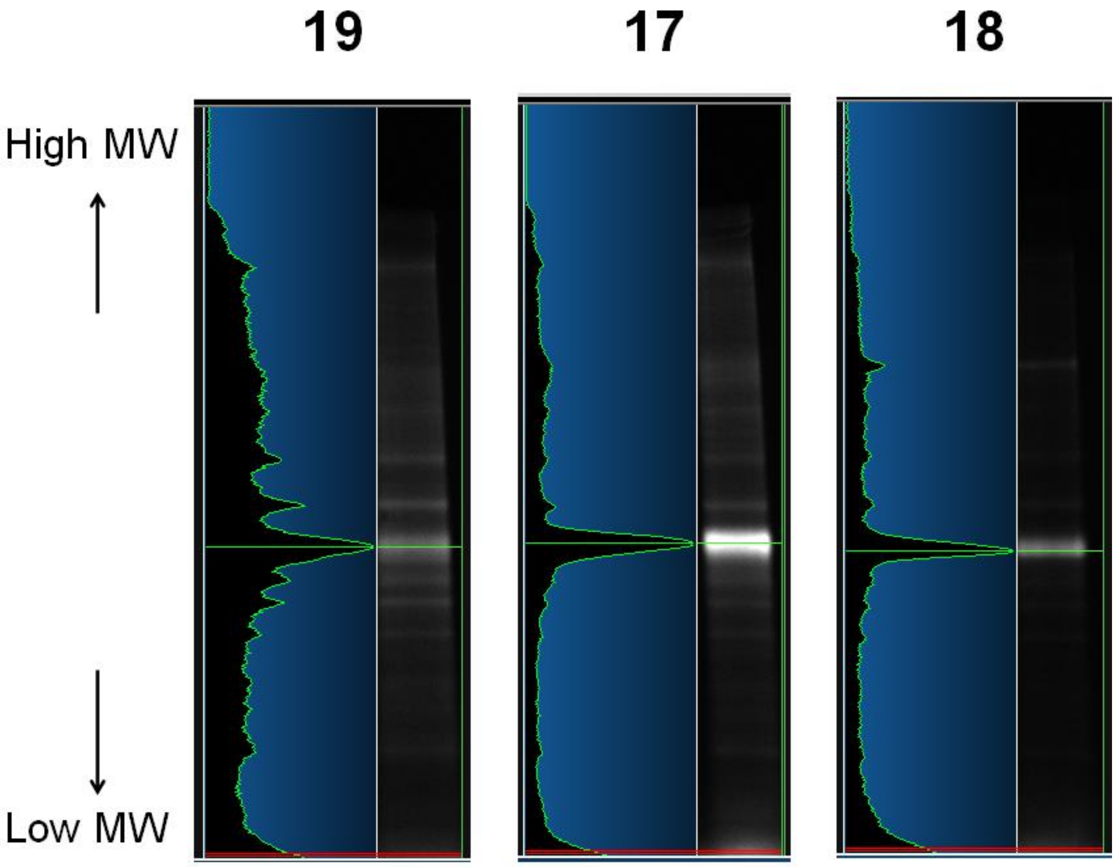

Figures S7. Selectivity of GST-HaloTag labeling. The signal intensities of Cy5-labeled proteins in Figure 5B were analyzed. Left: signal intensities in each band. Right: Image of fluorescent bands.

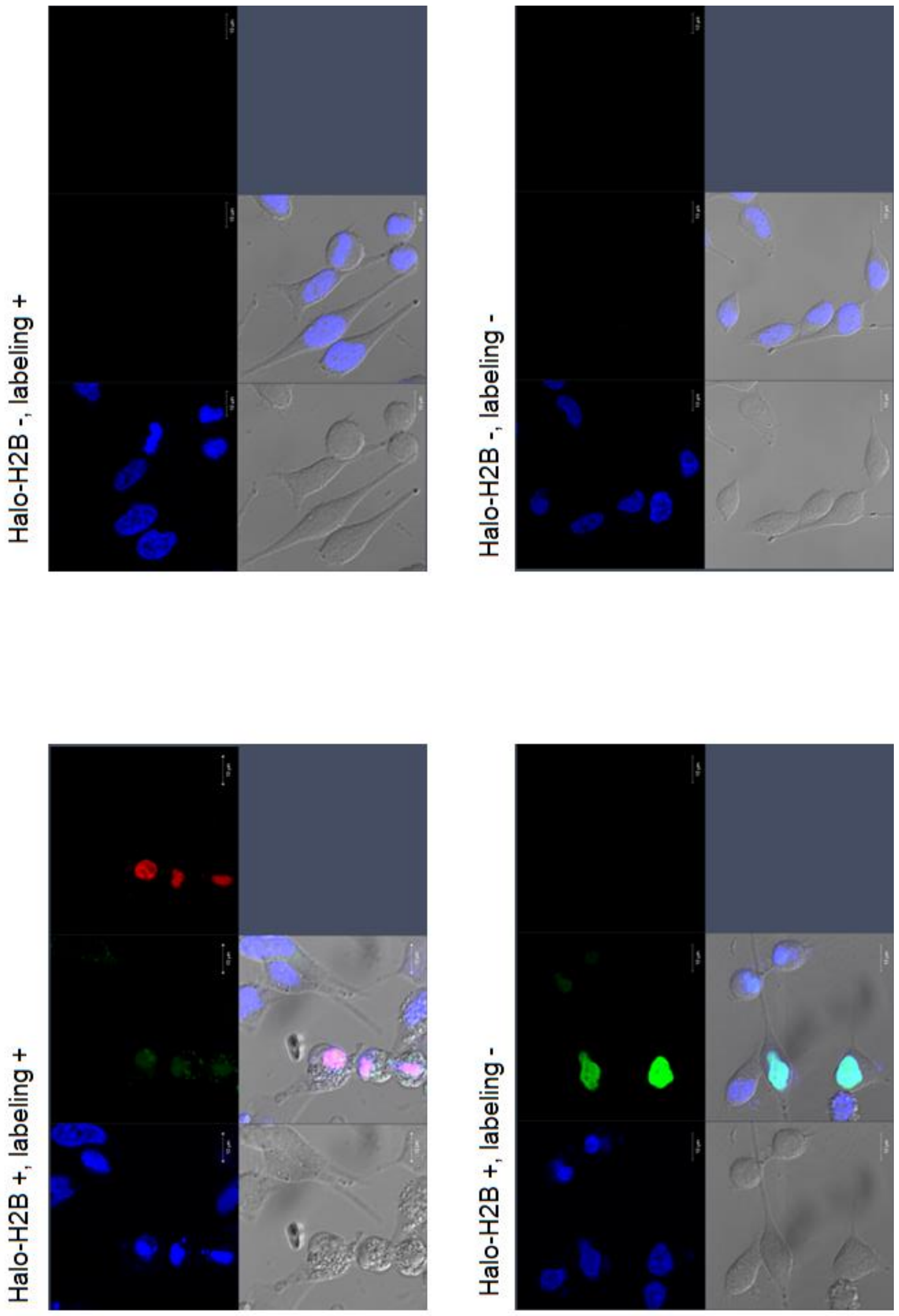

Figures S8. HaloTag-H2B Proximity histidine labeling using **18**. Green: **18**, blue: hoechest 33342, red: streptavidin-Texas Red

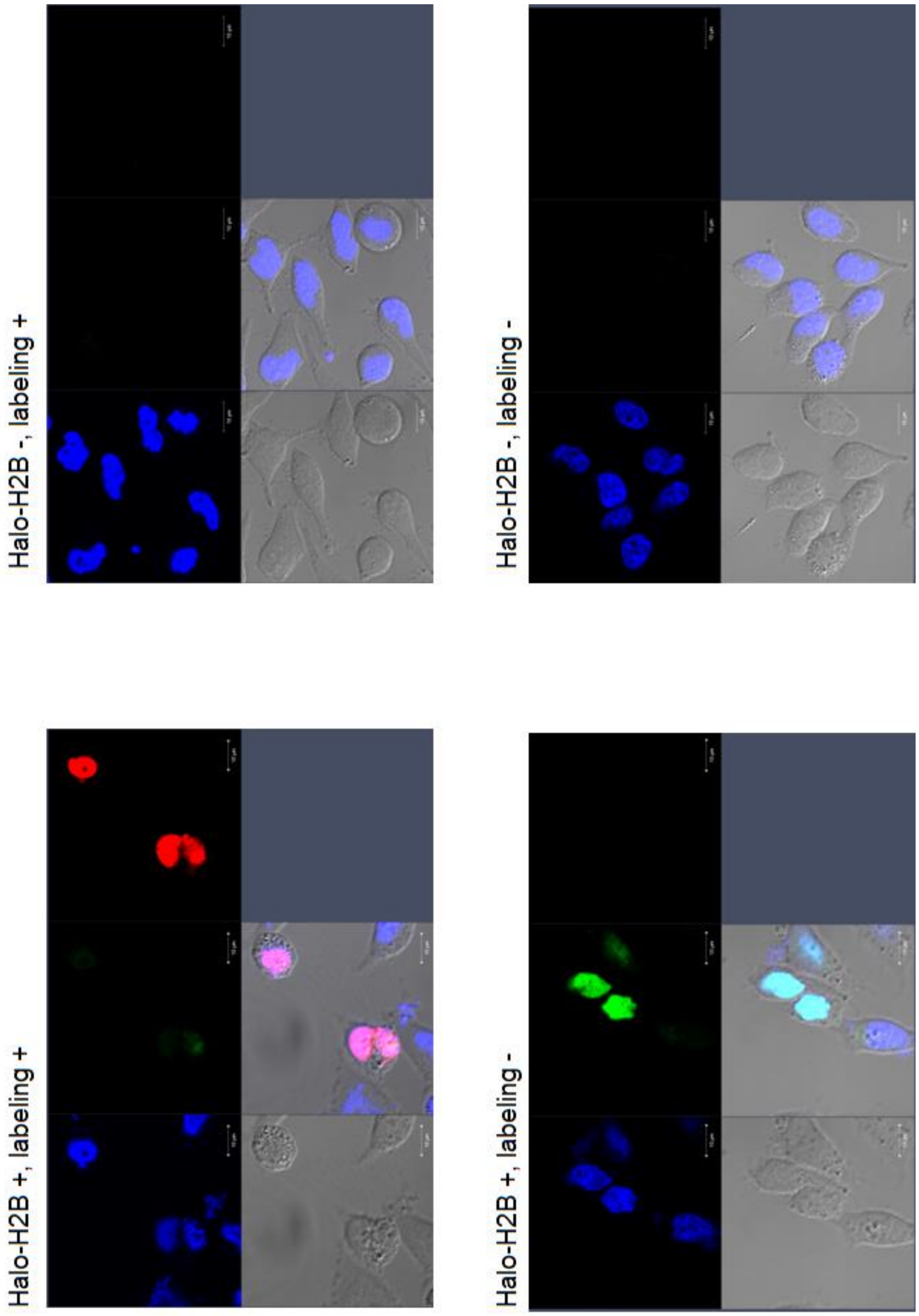

Figures S9. HaloTag-H2B Proximity histidine labeling using **19**. Green: **19**, blue: hoechest 33342, red: streptavidin-Texas Red

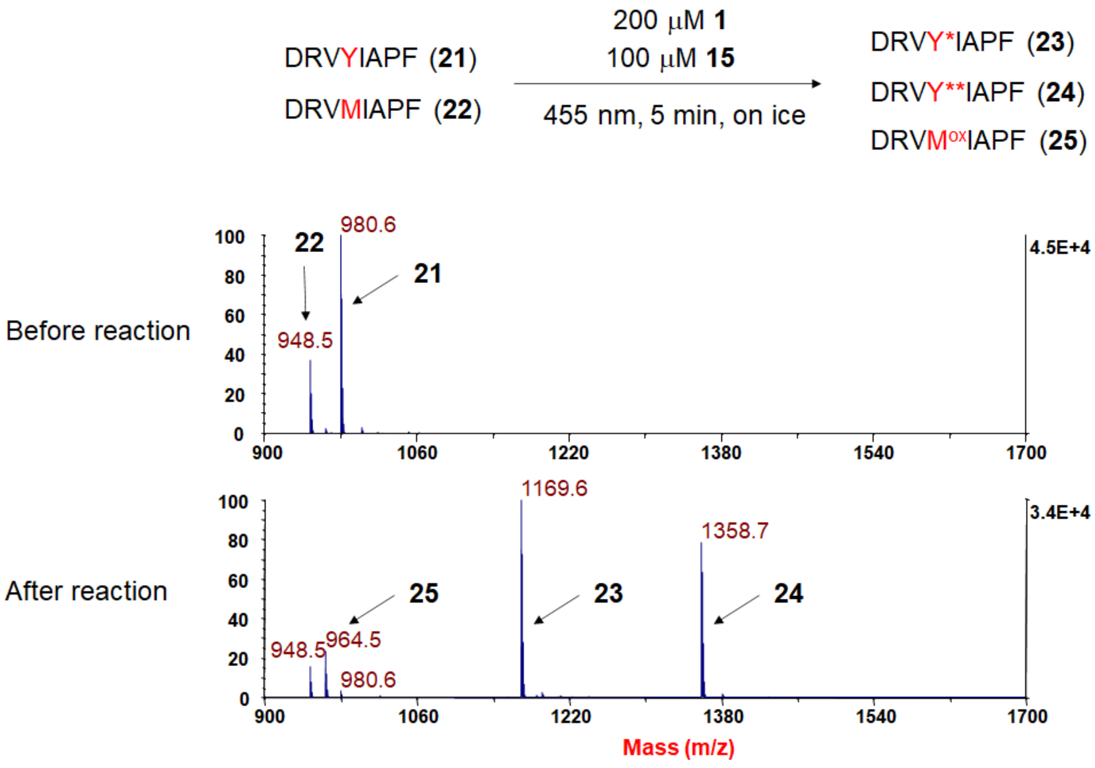

Figure S10. Reactions with two peptides DRV*Y*IAPF and DRV*M*IAPF under the reaction condition using **1** and **15**.

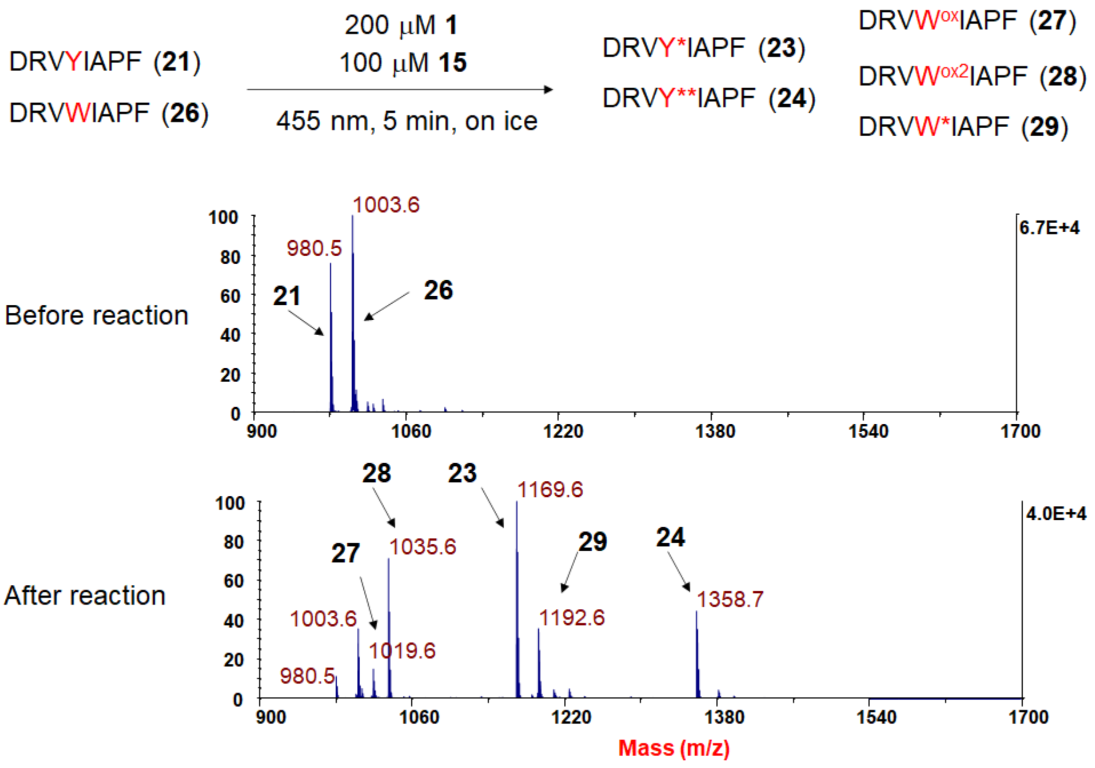

Figure S11. Reactions with two peptides DRV*Y*IAPF and DRV*W*IAPF under the reaction condition using **1** and **15**.

DRVY*IAPF (**23**)

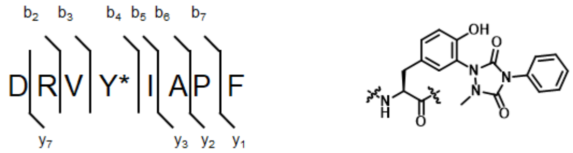

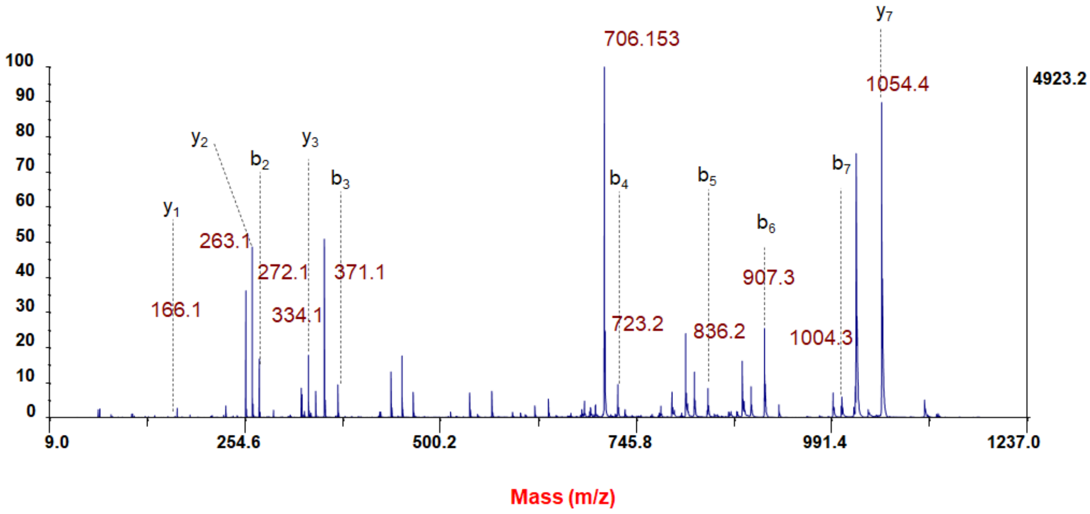

Figure S12. MS/MS analysis of DRVY*IAPF.

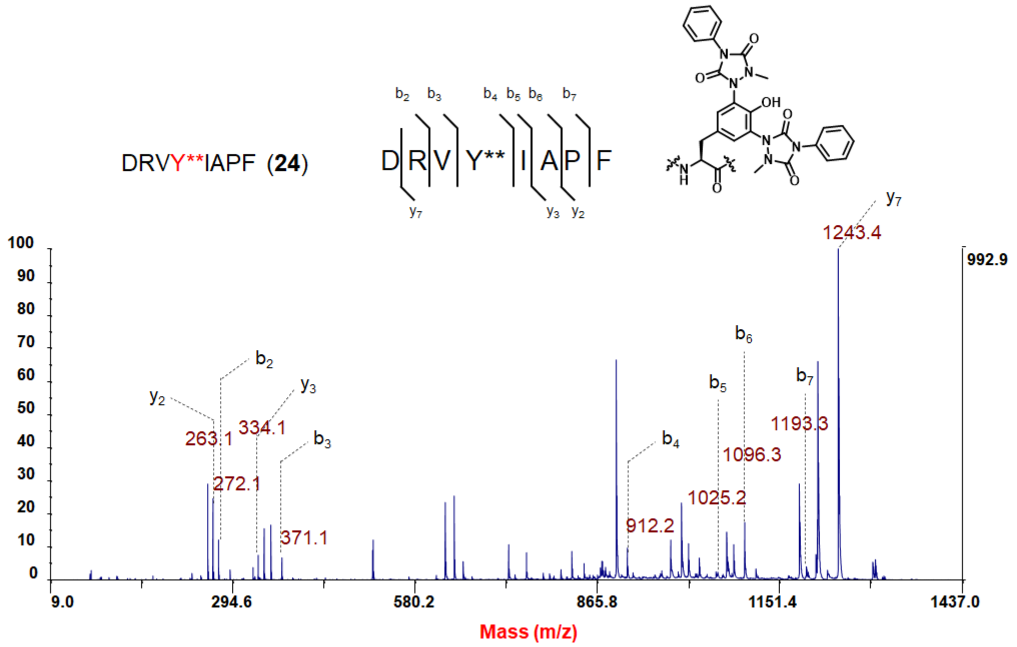

Figure S13. MS/MS analysis of DRVY**IAPF.

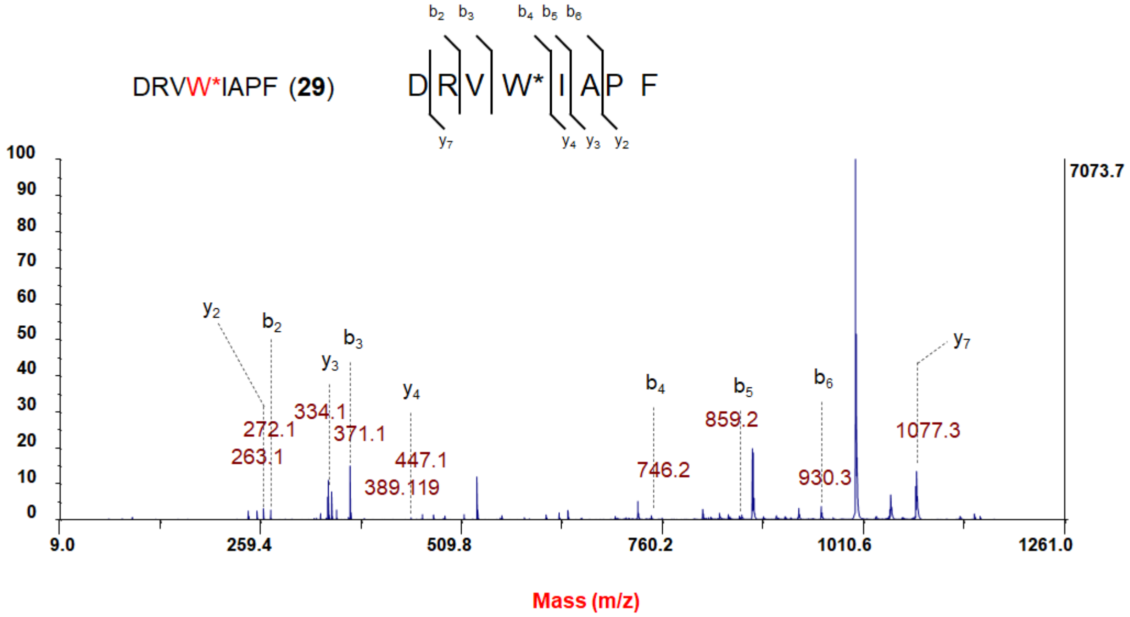

Figure S14. MS/MS analysis of DRVW*IAPF.
